# Diet quality is inversely associated with obesity in Chinese adults with type 2 diabetes

**DOI:** 10.1186/s12937-018-0374-6

**Published:** 2018-07-03

**Authors:** Lorena T. F. Cheung, Ruth S. M. Chan, Gary T. C. Ko, Eric S. H. Lau, Francis C. C. Chow, Alice P. S. Kong

**Affiliations:** 1 Department of Medicine and Therapeutics, The Chinese University of Hong Kong, Prince of Wales Hospital, Hong Kong SAR, China; 2 Centre for Nutritional Studies, The Chinese University of Hong Kong, Hong Kong SAR, China; 3 Li Ka Shing Institute of Health Sciences, The Chinese University of Hong Kong, Hong Kong SAR, China; 4 Hong Kong Institute of Diabetes and Obesity, The Chinese University of Hong Kong, Hong Kong SAR, China

**Keywords:** Diet quality, Obesity, Type 2 diabetes, Chinese

## Abstract

**Background:**

Diet quality has been linked to obesity, but this relationship remains unclear in individuals with type 2 diabetes (T2D). The aim of this study is to examine the association between diet quality and obesity in Chinese adults with T2D.

**Methods:**

Between April and November 2016, a total of 211 Chinese T2D adults who underwent assessment of diabetes-related treatment goals and metabolic control were recruited into two groups based on their body mass index (BMI): obese group (BMI ≥30 kg/m^2^) and non-obese group (BMI = 18.5–24.9 kg/m^2^). Diet quality indices including Alternate Healthy Eating Index-2010 (AHEI-2010), Diet Quality Index-International (DQI-I), and Dietary Approach to Stop Hypertension (DASH) score, were derived from a validated food frequency questionnaire.

**Results:**

Obese T2D patients had significantly lower AHEI-2010 (*P* < 0.001), DQI-I (*P* < 0.001), and DASH total scores (*P* = 0.044) than their non-obese counterparts, independent of age and sex. They also had higher total energy (*P* < 0.001), protein percentage of energy (*P* = 0.023), and meat, poultry and organ meat (*P* < 0.001), but lower vegetable (*P* = 0.014) intakes. Our multivariate logistic regression analyses demonstrated that the AHEI-2010, but not DQI-I and DASH, total score had an inverse association with obesity, independent of sociodemographics, anti-diabetic medication use, physical activity level and total energy intake (odds ratio [OR] per standard deviation (1-SD) increase: 0.95, 95% confidence interval [CI]: 0.91–0.99, *P* = 0.020). This association remained significant after further adjustment for glycemic control. Inverse associations were also found between obesity and multivariate-adjusted component scores, including AHEI-2010 red/processed meat (OR per 1-SD: 0.71, 95% CI: 0.51–0.99, *P* = 0.044), DQI-I variety (OR per 1-SD: 0.63, 95% CI: 0.46–0.86, *P* = 0.004), and DASH red/processed meat (OR per 1-SD: 0.57, 95% CI: 0.38–0.84, *P* = 0.005).

**Conclusions:**

Better diet quality, as characterized by higher AHEI-2010 scores, was associated with lower odds of obesity in Chinese adults with T2D. Dietary patterns reflecting high consumption of plant-based foods and low consumption of animal-based, high-fat, and processed foods may be imperative to optimize nutritional guidance for obesity management in this population.

**Electronic supplementary material:**

The online version of this article (10.1186/s12937-018-0374-6) contains supplementary material, which is available to authorized users.

## Background

Type 2 diabetes (T2D) is an alarming epidemic globally and of particular concern in Asia [1]. This epidemic is rapidly increasing in prevalence and presents unique clinical features among Asians, such as onset at a younger age and a lower body mass index (BMI), and more severe diabetic complications in younger patients and those exposed to famine prenatally or during early childhood [2, 3]. Diverse Asian populations, including Chinese population, are progressively adopting lifestyle factors characteristic of Western countries (e.g., diets higher in fat and sugar and lower in plant-based foods, combined with less physical activity), which further contributes to the marked increase in the rates of T2D in this region [4–7]. China now has the largest number of individuals affected by diabetes in any country, posing many challenges and a tremendous burden on its healthcare system due to diabetes-related complications and mortality [8]. As of the latest epidemiological study, which was conducted in 2013 and comprised 170,287 participants, 10.6% of the population of 1.38 billion people in China was diabetic, and 15.4 and 21.1% were overweight and obese, respectively [9]. In addition, the Hong Kong Diabetes Registry, an ongoing prospective cohort established in 1995, showed that obese T2D patients not only had worse metabolic control than T2D patients of normal weight but were also challenging to treat and had an extremely high risk of future diabetes-related cardiovascular-renal complications [10, 11].

Nutrients are not consumed in isolation in the daily diet. Thus, the analysis of dietary patterns of food consumption offers a holistic approach to disease prevention and treatment by addressing the collective health benefits of the whole diet and enhancing the practicality in public education and clinical application [12]. Previous dietary studies [13–16] and systematic reviews [17, 18] have reported that high compliance with health-promoting dietary patterns, characterized and quantified using corresponding diet quality indices such as the Dietary Approach to Stop Hypertension (DASH) score and the Alternate Healthy Eating-2010 Index (AHEI-2010), were associated with a significant reduction in the risk of obesity, cardiovascular disease, cancer, T2D and all-cause mortality, primarily in general populations from Western countries. Additionally, the Diet Quality Index-International (DQI-I) was developed to examine concerns related to chronic diseases, undernutrition and cross-national comparisons of diet quality using national nutrition surveys of the United States and China [19]. This score was also previously linked to a decrease in the risk of developing non-alcoholic fatty liver disease [20] and T2D [21] in the Hong Kong Chinese general population.

Questions remain concerning how specific dietary patterns are related to obesity in individuals with T2D, particularly in the Chinese population, which is undergoing both substantial changes in lifestyle factors and dramatic increases in diabetes prevalence. An understanding of the diet quality of Chinese T2D patients with and without obesity would contribute to the early detection of dietary characteristics that increase the likelihood of obesity and enable healthcare professionals to individualize effective dietary strategies for obesity prevention and treatment in this challenging population. We hypothesized that lower diet quality indices reflecting low adherence to health-promoting dietary patterns would be associated with an increased likelihood of obesity in Chinese T2D patients. The aim of this study was to investigate the association between diet quality, as assessed by AHEI-2010, DASH score and DQI-I, and obesity in Chinese T2D patients.

## Methods

### Study participants and setting

The present study was part of the cross-sectional analysis of the baseline data from an ongoing prospective study investigating lifestyle factors, including hedonic hunger, dietary characteristics and physical activity, and their relationships with glycemic and weight management in Chinese T2D patients [22]. In brief, patients with T2D who underwent regular comprehensive assessment for diabetes-related treatment targets and metabolic control at the Diabetes Mellitus and Endocrine Center of the Prince of Wales Hospital (PWH) between April and November 2016 were invited to participate. The PWH is a university-affiliated hospital with 1300 beds and 22 medical clinics serving a population of over 1.2 million, predominantly of Chinese ethnicity, in Hong Kong SAR, China. All participants had been diagnosed with T2D as defined by World Health Organization (WHO) criteria [23] and referred for assessment by their general practitioners and the hospital’s general medical and specialist clinics.

In this study, we recruited two groups of patients based on their body mass index (BMI): (1) an obese (BMI ≥ 30 kg/m^2^) group and (2) a non-obese (BMI = 18.5–24.9 kg/m^2^) comparison group. The participants were assigned to groups according to the WHO international classification of weight [24]. Additional eligibility criteria included age 18 to 65 years, Chinese ethnicity, and a documented diagnosis of T2D ≥ six months. The study excluded patients with type 1 diabetes, those who were pregnant or breastfeeding, and those with health conditions that may affect habitual dietary intake, such as end-stage renal failure (ESRF) requiring dialysis, chronic kidney disease (CKD) stage 4 and above, malignancy diagnosed within three years, and previous bariatric surgery. The study protocol was reviewed and approved by the Clinical Research Ethics Committee of The Chinese University of Hong Kong-Prince of Wales Hospital (CREC reference number 2016.126). All subjects provided informed written consent before participating in the study. The study was conducted in accordance with the Declaration of Helsinki.

### Assessment of clinical characteristics and diabetes-related metabolic control

The structured assessment of clinical characteristics and diabetes-related metabolic control, including anthropometric measurements, biochemical evaluations, and detailed documentation of socio-demographic data, medical history, and current medication use, was performed by trained diabetes nurses according to the modified European DiabCare protocol [25]. Standing height was assessed to the nearest 0.1 cm. Body weight was measured with a calibrated digital electric scale to the nearest 0.1 kg. BMI was calculated as weight in kilograms divided by height in squared meters (kg/m^2^). Waist circumference (WC) was determined at the approximate midpoint between the lower margin of the last palpable rib and the top of the iliac crest. Hip circumference was measured at the widest portion of the buttock over light clothing using a stretch-resistant plastic measuring tape. Systolic blood pressure (SBP) and diastolic blood pressure (DBP) were measured after the patient had rested in the sitting position for five to ten minutes. Blood samples were collected from the participants to test fasting plasma glucose (FPG), glycated hemoglobin (HbA_1c_), total cholesterol (TC), low-density lipoprotein cholesterol (LDL-C), high-density lipoprotein cholesterol (HDL-C) and triglycerides (TG) after the participants had fasted for at least eight hours. All biochemical analyses were performed according to the manufacturer’s specifications using standard protocols by the Department of Chemical Pathology laboratory at PWH.

### Assessment of physical activity level

An experienced dietitian conducted in-person interviews to assess the participants’ physical activity level using the locally validated International Physical Activity Questionnaire-Short Form (IPAQ-SF)–Chinese version [26]. The participants were asked to report their duration (minutes) and frequency (days) of walking, moderate-intensity activity, and vigorous-intensity activity performed for at least 10 min per session, during the previous seven days. The number of reported minutes per week in each category was converted to metabolic equivalents (METs) to obtain physical activity estimates independent of body weight and were expressed as MET-minutes/week.

### Assessment of dietary intake and diet quality

The same dietitian interviewed all participants face-to-face to assess their usual dietary intake over the past six months using the locally validated semi-quantitative food frequency questionnaire (FFQ) [27], and cross-checked the reported food intake with a diet history using a standard method. The FFQ contained 288 food items in eleven broad categories: cereals (including bread, pasta, and rice), vegetables, fruits, meat and poultry, fish and seafood, eggs, milk and dairy products, beverages, snacks, soups, and lastly, oils and condiments. Food models and common household utensils were used to facilitate portion size estimation. The amount of cooking oil used was calculated based on typical cooking methods and on the amount of cooking oil and the food portions reported by the participants [28]. Mean daily nutrient and food-group intakes were estimated using Food Processor® Nutrition Analysis and Fitness software version 8.0 (ESHA Research, Salem, Oregon, USA), which was supplemented with nutrient data on Chinese food items [29–31]. Macronutrient intakes, including protein, carbohydrates, total fat, saturated fatty acids (SFA), monounsaturated fatty acids (MUFA), and polyunsaturated fatty acids (PUFA), are presented as percentages of total energy intake, whereas other nutrient and food-group intakes are expressed on a per-1000-kcal basis to enable comparability across individuals with different total energy intakes [32].

The diet quality was assessed according to three established diet quality indices: AHEI-2010, DASH score and DQI-I. The scoring criteria of these diet quality indices used for the study participants are detailed in Additional file 1: Table S1-S3.

#### Alternate Healthy Eating Index-2010 (AHEI-2010)

We calculated the AHEI-2010 using the method described by Chiuve et al. [14], which consists of eleven dietary components with a score range of 0–10 for each component. A higher component score represents greater consumption of vegetables, fruit, whole grains, nuts and legumes, omega-3, and polyunsaturated fatty acid (PUFA) as well as a lower consumption of sugar-sweetened beverages and fruit juice, red and processed meat, trans fat, and sodium. A score of 0, 2.5, and 10 reflects the high, low, and moderate alcohol intakes, respectively. The overall AHEI-2010 score is the sum of the 11 dietary component scores, ranging from 0 (minimum score) to 110 (maximum score). A higher AHEI-2010 score indicates greater compliance with the Dietary Guidelines for Americans, which had been updated with additional dietary components that are predictive of chronic disease risk [14, 15], and therefore a higher diet quality.

#### Dietary Approach to Stop Hypertension (DASH) score

Adherence to the DASH diet was determined by a scale developed by Fung et al. [13]. The scale is based on eight food-group or nutrient components that are emphasized or minimized in the DASH diet, which encourages high intakes of whole grains, fruits, vegetables, nuts and legumes and low-fat dairy products but low intakes of sodium, sweetened beverages, and red and processed meats. For each component, all participants were classified into quintiles or quartiles according to the ranking of their intakes. Individuals with the highest quintile of intakes of fruits, vegetables, nuts and legumes were given a score of 5, while those with the lowest quintile of these intakes were given a score of 1. Conversely, the participants with the highest quintile intakes of sodium and red and processed meats received a score of 1, while those participants with the lowest quintile of intakes were given a score of 5. As noticeable numbers of the participants (52, 69, and 41% of this study cohort) had zero intake of whole grains, low-fat dairy products, and sweetened beverages, respectively, a modified scoring method for these components was adopted based on Bia et al. [33]. Participants were given a score of 1 for zero intake of whole grains and low-fat dairy products and a score of 5 for zero intake of sweetened beverages. The remaining participants were divided into quartiles. Subsequently, the participants with the lowest quartile of whole grain and low-fat dairy product consumption were given a score of 2, while those with the highest quartile of for the consumption of these products received a score of 5. A reverse-scoring method was used for sweetened beverages. The overall DASH score was the sum of the scores of eight dietary components, ranging from 8 (lowest adherence) to 40 (highest adherence).

#### Diet Quality Index-International (DQI-I)

The DQI-I was computed using the method by Kim et al. [19], with modifications from Chan et al. [20]. This diet quality index assesses four major aspects of diet quality: variety, adequacy, moderation, and overall balance of the diet, each of which has subcomponents. As the present study did not have adequate information to calculate the category of empty-calorie foods under the aspect ‘moderation’, the range of score for ‘moderation’ was adjusted to 0 to 24 instead of 0 to 30, and the DQI-I total score was adjusted from 0 to 94 instead of from 0 to 100, as proposed in the original method. A higher DQI-I score represents a higher-quality diet with better variety, adequacy, moderation, and overall balance.

### Statistical analysis

The variables compared between the non-obese and obese T2D groups included socio-demographic, clinical, and biochemical characteristics, physical activity level, total and component scores of diet quality indices (AHEI-2010, DQI-I, and DASH score), as well as nutrient and food-group intakes. Continuous variables following normal Gaussian distribution were expressed as mean ± standard deviation (SD) and compared by Independent Student’s t-test, whereas those with a skewed distribution were expressed as median (interquartile range) and compared with Wilcoxon rank-sum test. Categorical variables were expressed as number (percentage), and chi-square tests were performed for between-group comparisons of categorical variables. The analysis of covariance (ANCOVA) was used for between-group comparisons for continuous variables, and logistic regression was performed for categorical variables with adjustment for age and sex as covariates. The associations between diet quality and BMI and obesity status were examined by linear regression and logistic regression models, respectively, according to each SD increase in each diet quality index score. These analysis models were adjusted for covariates that were chosen based on their putative and potential influence on weight. The first model analyzed the crude data only. The second model was adjusted for age, sex, education level, employment status, smoking status, alcohol status, duration of diabetes, oral antidiabetic agent use, insulin use and physical activity level. The third model was further adjusted for total energy intake. The final model was also adjusted for HbA_1c_.

To estimate the sample size (n), we posed the following null hypothesis: there is no statistically significant difference in diet quality, as assessed by various diet quality indices, between obese and non-obese T2D patients. Most published literature reported that the mean ± SD values for the AHEI-2010, DASH and DQI-I were approximately 50 ± 11, 45 ± 8 and 23 ± 5, respectively. We set a 10% difference in the diet quality indices as the clinically relevant difference (*δ*). As the ‘standardized difference’ was the ratio of *δ* and SD to various diet quality indices, the corresponding ‘standardized differences’ were calculated as 5.0/11 (=0.45), 4.5/8 (=0.56) and 2.3/5 (=0.46), respectively.

With ‘standardized differences’ of 0.45–0.56, a statistical power of 90% and a significance level of 5%, a minimum sample size (n) = 140–200 was estimated using Altman’s nomogram for calculating sample size [34]. Hence, each group required 70 to 100 patients. With extra 5% added as a consideration for potential incomplete data collection after subject recruitment, a sample size of (n) = 105 or above was estimated for each of the two patient groups. All statistical analyses were performed using SPSS software Version 23 (2015, SPSS, Inc., Chicago, USA). A *P*-value < 0.05 (2-tailed) was used to denote statistically significant differences.

## Results

### Study participant characteristics

Table 1 summarizes the socio-demographic, clinical, and biochemical characteristics and the physical activity level of the study participants. This study enrolled a total of 211 Hong Kong Chinese adults with T2D (115 men and 96 women), of whom 105 (49.8%) and 106 (50.2%) were in the non-obese and obese groups, respectively. The obese T2D patients were younger and took more antihypertensive and lipid-lowering drugs. There were no significant between-group differences in socio-demographics, duration of diabetes, oral antidiabetic drug use, insulin use and glycemic profile. Compared with the non-obese T2D group, the obese T2D group displayed significantly worse blood pressure and lipid profiles, including higher DBP (*P* = 0.004) and TG (*P* = 0.007), but lower HDL-C (*P* < 0.001), independent of age and sex. The physical activity level was also lower in the obese T2D group, although the difference was not significant.

**Table 1:** Characteristics of the Chinese type 2 diabetic adults with and without obesity

	All (*n* = 211)	Non-obese T2D (*n* = 105)	Obese T2D (*n* = 106)	*P*-value	*P*-value adjusted for age and sex
Social-demographics					
Age, years	54.0 ± 8.6	55.8 ± 7.5	52.1 ± 9.2	0.001	–
Sex					
Male	115 (54.5)	57 (49.6)	58 (50.4)	0.950	–
Female	96 (45.5)	48 (50.4)	48 (49.6)		
Weight, kg	78.1 ± 21.1	61.2 ± 8.9	94.8 ± 15.7	< 0.001	< 0.001
BMI, kg/m^2^	28.7 ± 6.8	22.7 ± 1.6	34.6 ± 4.3	< 0.001	< 0.001
Waist circumference, cm	97.6 ± 16.2	84.3 ± 7.4	110.7 ± 11.1	< 0.001	< 0.001
Waist-hip ratio	0.95 ± 0.07	0.91 ± 0.06	0.98 ± 0.07	< 0.001	< 0.001
Education level, secondary or above	160 (75)	78 (74.3)	82 (77.4)	0.602	0.415
Full/Part-time employment	136 (64.4)	66 (62.9)	70 (66.0)	0.629	0.446
Smoking	50 (23.7)	24 (22.9)	26 (24.5)	0.775	0.665
Alcohol drinking	5 (2.4)	3 (2.9)	2 (1.9)	0.643	0.696
Clinical and biochemical profile					
History of hypertension	146 (69.2)	59 (56.2)	87 (82.1)	< 0.001	< 0.001
History of cardiovascular disease	39 (18.5)	21 (20.0)	18 (17.0)	0.572	0.810
Duration of diabetes, years	8.8 ± 7.7	9.1 ± 7.8	8.5 ± 7.6	0.566	0.804
Oral antidiabetic drug use	188 (89.1)	93 (88.6)	95 (89.6)	0.806	0.439
Insulin use	72 (34.1)	31 (29.5)	40 (37.7)	0.266	0.331
Antihypertensive drug use	142 (67.3)	58 (55.2)	84 (79.2)	< 0.001	< 0.001
Lipid-lowering drug use	134 (63.5)	57 (54.3)	77 (72.6)	0.006	< 0.001
SBP, mmHg	131.5 ± 20.3	130.1± 21.8	132.9 ± 18.6	0.311	0.186
DBP, mmHg	74.5 ± 11.7	72.0 ± 11.1	77.1 ± 11.7	< 0.001	0.004
FPG, mmol/L	7.6 ± 2.7	7.5 ± 2.2	7.7 ± 2.3	0.601	0.438
HbA_1C_, %	7.7 ± 1.5	7.6 ± 1.6	7.7 ± 1.4	0.495	0.222
TC, mmol/L	4.14 ± 0.86	4.21 ± 0.85	4.07 ± 0.87	0.274	0.211
LDL-C, mmol/L	2.19 ± 0.74	2.23 ± 0.78	2.15 ± 0.70	0.439	0.334
HDL-C, mmol/L	1.25 ± 0.36	1.34 ± 0.39	1.15 ± 0.30	< 0.001	< 0.001
TG, mmol/L	1.33 (0.93–1.97)	1.12 (0.79–1.77)	1.42 (1.06–2.08)	0.033	0.007
Physical activity					
Physical activity level, MET-minutes/week	2088.2 ± 1642.1	2202.8 ± 1789.0	1974.6 ± 1482.1	0.314	0.476

Variables are presented as mean ± standard deviation, median (interquartile) or frequency (percentage) (%)

*BMI* body mass index, *DBP* Diastolic blood pressure, *FPG* fasting plasma glucose, *HbA*_*1C*_ glycated hemoglobin, *HDL-C* high-density lipoprotein, *LDL-C* low-density lipoprotein cholesterol, *SBP* Systolic blood pressure, *T2D* type 2 diabetes, *TC* total cholesterol, *TG* triglyceride

### Diet quality indices

The total and component scores of the diet quality indices for the study participants are shown in Table 2. The obese T2D group had a significantly lower age- and sex-adjusted AHEI-2010 total (57.8 ± 11.3 vs 63.1 ± 12.2, *P* < 0.001) and red/processed meat component scores (2.4 ± 2.7 vs 3.9 ± 2.8, *P* < 0.001), DQI-I total scores (51.7 ± 7.0 vs 54.3 ± 7.8, *P* < 0.001), variety (17.9 ± 1.6 vs 18.6 ± 1.5, *P* = 0.001), moderation (7.5 ± 4.6 vs 9.3 ± 5.0, *P* = 0.029) component scores and DASH total (21.7 ± 4.8 vs 23.5 ± 5.4, *P* = 0.044) and red/processed meat component scores (2.6 ± 1.4 vs 3.5 ± 1.3, *P* < 0.001) compared with their non-obese counterparts, indicating worse diet quality in the obese T2D group (Table 2).

**Table 2:** Total and component scores of the diet quality indices of the Chinese type 2 diabetic adults with and without obesity

Dietary component (score range)	All (*n* = 211)	Non-obese T2D (*n* = 105)	Obese T2D (*n* = 106)	*P*-value	*P*-value adjusted for age and sex
AHEI-2010^a^					
Total score (0–110)	60.4 ± 12.0	63.1 ± 12.2	57.8 ± 11.3	0.001	< 0.001
Component scores					
Vegetables (0–10)	4.4 ± 2.0	4.6 ± 2.1	4.2 ± 1.9	0.151	0.113
Fruits (0–10)	3.2 ± 1.4	3.2 ± 1.4	3.1 ±1.4	0.598	0.625
Whole grains (0–10)	3.9 ± 4.5	4.5 ± 4.5	3.3 ± 4.4	0.050	0.056
Sugar-sweetened beverages and fruit juice (0–10)	7.1 ± 4.1	7.4 ± 3.8	6.9 ± 4.3	0.446	0.625
Nuts and legumes (0–10)	7.3 ± 3.2	7.3 ± 3.1	7.4 ±3.5	0.889	0.536
Red/processed meat (0–10)	3.1 ± 2.9	3.9 ± 2.8	2.4 ± 2.7	< 0.001	< 0.001
Trans fat, % of total energy (0–10)	6.2 ± 3.4	6.4 ± 3.1	6.0 ± 3.6	0.373	0.604
Long-chain omega-3 fats (EPA + DHA) (0–10)	9.2 ± 2.1	9.5 ± 1.5	8.9 ± 2.5	0.027	0.119
PUFA, % of total energy (0–10)	7.9 ± 2.2	7.9 ± 2.1	7.9 ± 2.2	0.997	0.730
Sodium (0–10)	5.3 ± 2.9	5.4 ± 3.1	5.1 ± 2.7	0.431	0.439
Alcohol (0–10)	2.8 ± 1.5	3.0 ± 1.9	2.6 ±1.0	0.087	0.153
DQI-I^a^					
Total score (0–94)	53.0 ± 7.5	54.3 ± 7.8	51.7 ± 7.0	0.012	< 0.001
Component scores					
Variety (0–20)	18.3 ± 1.6	18.6 ± 1.5	17.9 ± 1.6	0.002	0.001
Adequacy (0–30)	25.6 ± 5.3	25.6 ± 5.7	25.5 ± 5.0	0.882	0.948
Moderation (0–24)	8.4 ± 4.9	9.3 ± 5.0	7.5 ± 4.6	0.010	0.029
Overall balance (0–10)	0.7 ± 1.3	0.8 ± 1.4	0.7 ± 1.2	0.576	0.460
DASH score^a^					
Total score (8–40)	22.6 ± 5.2	23.5 ±5.4	21.7 ± 4.8	0.016	0.044
Component scores					
Fruits (1–5)	3.0 ± 1.4	3.0 ±1.4	3.0 ± 1.3	0.962	0.846
Vegetables (1–5)	3.0 ± 1.4	3.1 ± 1.4	3.0 ± 1.4	0.626	0.638
Nuts and legumes (1–5)	3.1 ± 1.4	3.0 ±1.4	3.1 ± 1.4	0.530	0.246
Sodium (1–5)	3.1 ± 1.4	3.2 ± 1.5	3.0 ± 1.4	0.335	0.297
Red and processed meat (1–5)	3.1 ± 1.4	3.5 ± 1.3	2.6 ± 1.4	< 0.001	< 0.001
Whole grains (1–5)	2.3 ± 1.6	2.4 ± 1.6	2.1 ± 1.5	0.099	0.123
Low-fat dairy (1–5)	1.8 ± 1.3	1.9 ± 1.4	1.7 ± 1.3	0.171	0.141
Sweetened beverages (1–5)	3.5 ± 1.6	3.5 ± 1.6	3.4 ± 1.6	0.617	0.952

Variables are presented as mean ± standard deviation

^a^A higher total diet quality index score indicates a higher diet quality

*AHEI-2010* Alternate Healthy Eating Index-2010, *DHA* docosahexaenoic acid, *DASH* Dietary Approaches to Stop Hypertension score, *DQI-I* Diet Quality Index-International, *EPA* eicosapentaenoic acid, *PUFA* polyunsaturated fatty acids

### Daily nutrient and food-group intakes

The daily nutrient and food-group intakes of the non-obese and obese Chinese adults with T2D are presented in Table 3. The obese T2D patients consumed significantly higher total energy amounts (*P* < 0.001) and percentages of protein from total energy (*P* = 0.023) than the non-obese adults with T2D, independent of age and sex. On an energy-adjusted basis, the intakes of beneficial nutrients and food groups, including dietary fiber, vitamin C, calcium, potassium, magnesium, and vegetables, were significantly lower in the obese T2D group, whereas the intakes of meat, poultry, and organ meat were higher (all *P* < 0.05).

**Table 3:** Daily nutrient and food-group intakes of the Chinese type 2 diabetic adults with and without obesity

	Total (*n* = 211)	Non-obese T2D (*n* = 105)	Obese T2D (*n* = 106)	*P*-value	*P*-value adjusted for age and sex
Total energy intake, kcal/day	2024.6 ± 514.6	1877.4 ± 441.3	2154.4 ± 546.0	< 0.001	< 0.001
Protein, % of total energy	19.2 ± 3.0	18.7 ± 2.5	19.6 ± 3.3	0.018	0.023
Carbohydrates, % of total energy	41.8 ± 7.0	42.8 ± 7.0	40.9 ± 6.6	0.035	0.070
Total fat, % of total energy	39.1 ± 5.4	38.7 ± 5.5	39.4 ± 5.3	0.291	0.438
SFA, % of total energy	8.1 ± 1.8	7.9 ± 1.9	8.2 ± 1.7	0.183	0.480
MUFA, % of total energy	15 ± 3.4	14.6 ± 3.6	15.4 ± 3.2	0.089	0.161
PUFA, % of total energy	9.5 ± 3.0	9.5 ± 3.0	9.5 ± 2.8	0.918	0.907
Trans fat, g*	2.1 ± 2.0	2.0 ± 2.0	2.2 ± 2.0	0.644	0.683
Cholesterol, mg*	171.1 ± 50.8	166.1 ± 50.6	176.1 ± 50.8	0.152	0.189
Dietary fiber, g*	6.6 ± 2.6	7.2 ± 2.8	6.0 ± 2.3	0.001	0.002
Vitamin C, mg*	52.8 ± 29.6	58.0 ± 33.2	47.6 ± 24.8	0.010	0.016
Calcium, mg*	283.8 ± 108.1	302.8 ± 103.6	264.9 ± 109.6	0.011	0.023
Phosphorus, mg*	598.4 ± 106.2	605.3 ± 105.8	591.7 ± 106.6	0.354	0.559
Sodium, mg*	1750.5 ± 444.4	1818 ± 440.5	1682.8 ± 440.0	0.026	0.061
Potassium, mg*	1095.1 ± 230.6	1132.5 ± 236.7	1058.0 ± 219.2	0.019	0.027
Iron, mg*	7.3 ± 2.4	7.5 ± 2.5	7.1 ± 2.3	0.228	0.525
Magnesium, mg*	140.0 ± 35.4	147.0 ± 39.1	133.0 ± 29.9	0.004	0.013
Zinc, mg*	5.1 ± 1.0	5.0 ± 0.9	5.2 ± 1.0	0.087	0.167
Refined grains, g*	205.3 ± 75.2	197.5 ± 77.9	213.0 ± 72.0	0.137	0.139
Whole grains, g*	38.2 ± 62.2	43.4 ± 62.6	33.1 ± 61.7	0.227	0.303
Vegetables, g*	96.5 ± 62.5	107.6 ± 71.6	85.4 ± 49.8	0.010	0.014
Fruits and dried fruits, g*	83 ± 49.9	88.5 ± 54.2	77.5 ± 44.9	0.108	0.162
Meat, poultry and organ meat, g*	72.9 ± 32.2	63.6 ± 28.4	82.1 ± 33.3	< 0.001	< 0.001
Fish and seafood, g*	33.5 ± 23	34.3 ± 22.8	32.7 ± 23.4	0.629	0.973
Egg and egg products, g*	11.8 ± 9.7	11.7 ± 9.8	12.0 ± 9.7	0.753	0.880
Milk and dairy products, ml*	20.7 ± 41.7	24.3 ± 43.1	17.1 ± 40.2	0.208	0.208
Fast food, g*	5.3 ± 8.9	5.4 ± 9.0	5.2 ± 8.7	0.881	0.291
Nuts, g*	2.8 ± 5.7	3.6 ± 6.4	2.1 ± 4.7	0.046	0.057
Sugar sweetened beverages, ml*	44.3 ± 67.1	42.3 ± 63.9	46.3 ± 70.4	0.515	0.890
Sweet pastries and desserts, g*	5.3 ± 10.7	4.2 ± 9.9	6.4 ± 11.3	0.135	0.129
Soups, ml*	70.3 ± 74.6	78.1 ± 76.3	62.7 ± 72.4	0.134	0.280
Alcohol, g*	0.6 ± 2.3	0.8 ± 2.5	0.4 ± 2.1	0.303	0.253
Cooking oils and fats, g*	20.0 ± 5.9	19.6 ± 5.1	20.5 ± 6.5	0.242	0.322

Variables are presented as mean ± standard deviation

MUFA: monounsaturated fatty acids, PUFA: polyunsaturated fatty acids, SFA, saturated fatty acids, T2D: type 2 diabetes

*per 1000 kcal^−1^ day^−1^

### Association between diet quality and BMI

Table 4 shows the beta (β) coefficients ± standard error (SE) of BMI according to one SD increase in each dietary quality index score from linear regression analysis. Those significant scores displayed in Table 2 were included in the univariate and multivariate models. There were no significant associations between AHEI-2010, DQI-I and DASH total scores, and BMI after multivariate adjustments (Model 2–4). Only AHEI red/processed meat (β ± SE = − 2.00 ± 0.53, *P* < 0.001), DQI-I variety (β ± SE = − 1.23 ± 0.47, *P* = 0.010) and moderation (β ± SE = − 1.40 ± 0.52, *P* = 0.008) and DASH red/processed meat (β ± SE = − 2.30 ± 0.52, *P* = 0.006) component scores were significantly and negatively associated with BMI, independent of socio-demographics, diabetes duration, antidiabetic medications and physical activity level (Model 2). The inverse associations for these component scores, except DQI-I variety score, remained significant after additional adjustments for total energy intake (Model 3) and glycemic control (Model 4).

**Table 4:** Beta coefficients (standard error) of body mass index according to each standard deviation increase in diet quality index scores from linear regression analysis

	Model 1^a^		Model 2^b^		Model 3^c^		Model 4^d^	
	Beta (SE)	*P*-value	Beta (SE)	*P*-value	Beta (SE)	*P*-value	Beta (SE)	*P*-value
AHEI-2010								
Total score^e^	− 1.09 (0.46)	0.019	− 0.95 (0.50)	0.060	− 0.54 (0.49)	0.272	−0.52 (0.49)	0.293
Red/processed meats^f^	− 1.81 (0.45)	< 0.001	−2.00 (− 0.53)	< 0.001	− 1.19 (0.59)	0.046	− 1.22 (0.59)	0.040
DQI-I								
Total score^g^	− 0.94 (0.46)	0.044	− 0.93 (0.49)	0.062	− 0.66 (0.48)	0.054	− 0.66 (0.48)	0.167
Variety^h^	− 1.05 (0.46)	0.025	−1.23 (0.47)	0.010	−0.96 (0.45)	0.036	−0.98 (0.46)	0.033
Moderation^i^	− 1.44 (0.46)	0.002	− 1.40 (0.52)	0.008	− 0.30 (0.59)	0.607	− 0.27 (0.59)	0.652
DASH score								
Total score^j^	− 0.87 (0.47)	0.063	− 0.79 (0.50)	0.120	− 0.26 (0.50)	0.599	− 0.24 (0.50)	0.272
Red/processed meats^k^	− 2.03 (0.45)	< 0.001	− 2.30 (0.52)	< 0.001	−1.60 (− 0.57)	0.006	− 1.63 (0.57)	0.005

^a^Model 1: crude data

^b^Model 2: adjusted for age, sex, education level, employment status, smoking status, alcohol drinking status, duration of diabetes, oral antidiabetic drug use, insulin use, physical activity level

^c^Model 3: adjusted for all factors in Model 2, plus total energy intake

^d^Model 4: adjust for all factors in Model 3, plus glycated hemoglobin

^e^1 SD = 12.0 points

^f^1 SD = 2.9 points

^g^1 SD = 7.5 points

^h^1 SD = 1.6 points

^i^1 SD = 4.9 points

^j^1 SD = 5.2 points

^k^1 SD = 1.4 points

*AHEI-2010* Alternate Healthy Eating Index-2010, *CI* confidence interval, *DASH* Dietary Approaches to Stop Hypertension score, *DQI-I* Diet Quality Index-International, *OR* odds ratio, *SE* standard error

### Association between diet quality and obesity

We performed logistic regression analyses to determine whether diet quality, as measured by AHEI-2010, DQI-I and DASH total and component scores, was associated with obesity status (Table 5). On the basis of per 1-SD increase in each diet quality index score, higher total AHEI total (odds ratio (OR): 0.64, 95% confidence interval (CI) 0.46, 0.88, *P* = 0.006) and red/processed meat component (OR: 0.51, 95% CI 0.36, 0.73, *P* < 0.001), DQI-I total (OR: 0.68, 95% CI 0.50, 0.93, *P* = 0.017), variety (OR: 0.60, 95% CI 0.44, 0.81, *P* = 0.001) and moderation component (OR: 0.69, 95% CI 0.50, 0.95, *P* = 0.024) scores as well as DASH total (OR: 0.71, 95% CI 0.52, 0.97, *P* = 0.032) and red/processed meat component (OR: 0.46, 95% CI 0.32, 0.66, *P* < 0.001) scores, were significantly associated with a lower likelihood of obesity, independent of socio-demographics, diabetes duration, antidiabetic medications and physical activity level (Model 2). However, such associations for these diet index scores were attenuated by additional adjustment of total energy intake, although AHEI-2010 total (OR: 0.95, 95% CI 0.91, 0.99, *P* = 0.020) and red/processed meat component (OR: 0.71, 95% CI 0.51, 0.99, *P* = 0.044), DQI-I variety component (OR: 0.63, 95% CI 0.46, 0.86, *P* = 0.004) as well as DASH red/processed meat component (OR: 0.57, 95% CI 0.38, 0.84, *P* = 0.005) scores remained significantly inverse-associated with obesity status (Model 3). No noticeable differences were observed upon further adjustment for glycemic control in the final model.

**Table 5:** Odds ratios (95% confidence interval) of obesity^a^ according to each standard deviation increase in diet quality index scores from logistic regression analysis

	Model 1^b^		Model 2^c^		Model 3^d^		Model 4^e^	
	OR (95% CI)	*P*-value	OR (95% CI)	*P*-value	OR (95% CI)	*P*-value	OR (95% CI)	*P*-value
AHEI-2010								
Total score^f^	0. 63 (0.47, 0.84)	0.002	0.64 (0.46, 0.88)	0.006	0.95 (0.91, 0.99)	0.020	0.95 (0.91, 0.99)	0.018
Red/processed meats^g^	0.57 (0.43, 0.76)	< 0.001	0.51 (0.36, 0.73)	< 0.001	0.71 (0.51, 0.99)	0.044	0.66 (0.44, 0.99)	0.042
DQI-I								
Total score^h^	0.70 (0.53, 0.93)	0.014	0.68 (0.50. 0.93)	0.017	0.73 (0.53, 1.01)	0.054	0.73 (0.53, 1.00)	0.052
Variety^i^	0.65 (0.49, 0.86)	0.003	0.60 (0.44, 0.81)	0.001	0.63 (0.46, 0.86)	0.004	0.62 (0.45, 0.94)	0.003
Moderation^j^	0.70 (0.53, 0.92)	0.030	0.69 (0.50, 0.95)	0.024	0.99 (0.68, 1.46)	0.977	0.99 (0.68, 1.48)	0.990
DASH score								
Total score^k^	0.71 (0.54, 0.94)	0.017	0.71 (0.52, 0.97)	0.032	0.83 (0.60, 1.15)	0.266	0.83 (0.60, 1.16)	0.272
Red/processed meats^l^	0.52 (0.39, 0.70)	< 0.001	0.46 (0.32, 0.66)	< 0.001	0.57 (0.38, 0.84)	0.005	0.56 (0.38, 0.84)	0.004

^a^Obesity is defined as body mass index (BMI) ≥30 kg/m^2^

^b^Model 1: crude data

^c^Model 2: adjusted for age, sex, education level, employment status, smoking status, alcohol drinking status, duration of diabetes, oral antidiabetic drug use, insulin use, physical activity level

^d^Model 3: adjusted for all factors in Model 2, plus total energy intake

^e^Model 4: adjust for all factors in Model 3, plus glycated hemoglobin

^f^1 SD = 12.0 points

^g^1 SD = 2.9 points

^h^1 SD = 7.5 points

^i^1 SD = 1.6 points

^j^1 SD = 4.9 points

^k^1 SD = 5.2 points

^l^1 SD = 1.4 points

*AHEI-2010* Alternate Healthy Eating Index-2010, *CI* confidence interval, *DASH* Dietary Approaches to Stop Hypertension score, *DQI-I* Diet Quality Index-International, *OR* odds ratio, *SD* standard deviation

## Discussion

To our knowledge, this study was the first to characterize the disparities in diet quality between obese Chinese T2D patients and their non-obese counterparts and to report an inverse association between diet quality and obesity in this population. AHEI-2010 total score had a weak, but significant, inverse association with obesity in Chinese T2D adults, independent of socio-demographics, anti-diabetic medications, total energy intake, physical activity level and glycemic control. Each SD increase in AHEI total score was associated with 5% of the reduced likelihood of being obese after controlling for these confounders. Furthermore, multivariate-adjusted AHEI-2010 (red/processed meats), DQI-I (variety) and DASH (red/processed meats) component scores were significantly and negatively associated with both BMI and obesity. Our findings may implicit the importance of attaining better dietary quality, including more dietary variety and reduced red/processed meat intake, in the obesity management of T2D patients.

The results of the present study in the Chinese T2D population are consistent with prior reports on the inverse relationship between diet quality, as assessed by AHEI-2010, DASH score and other diet quality indices, and obesity/BMI in the general populations from Western countries [16, 17, 35]. Furthermore, the DQI-I total scores of all non-obese and obese T2D patients in the present study were lower than those reported in earlier research by our group [21] for the general Hong Kong Chinese population with a lower mean BMI. This may imply lower variety, adequacy, moderation and overall balance in the diet of the T2D population, particularly those with obesity.

In addition to the DQI-I, another important diet quality index, the Chinese Diet Balance Index (DBI), was developed according to the Chinese dietary guidelines and the Chinese Food Pagoda [36]. This index has three indicators reflecting excessive food intake, deficient food intake and unbalanced food intake. Previous literature indicates that the Chinese DBI has been used to examine diet quality in different general populations, such as middle-aged and older adults [37] and pregnant women [38], and across seasons and areas of residence [39]. As our group previously used the DQI-I to evaluate the relationship between diet quality and the development of T2D in the general Hong Kong Chinese population [21], the same index was used in this study for comparison. However, we believe it will be important to further examine the use of the Chinese DBI in our Chinese T2D population in future studies.

Nutrition therapy has been recognized as the cornerstone of diabetes management by various diabetes organizations and health authorities in Western countries [40–42] and Asia [43–46]. There is a common consensus among nutritional guidelines worldwide regarding the recommendation of a reduced energy intake and a healthy lifestyle for overweight and obese adult diabetic patients. Our finding of higher age- and sex-adjusted energy intake by obese T2D patients compared with their non-obese counterparts conforms with the recommendation to reduce calories for weight reduction that is advocated by the current diabetes nutritional guidelines. However, most of these guidelines place greater emphasis on specific nutrients and nutrient levels than on dietary patterns for diabetes management. Earlier studies suggest that adherence to a specific dietary pattern that promotes metabolic health may be more beneficial for diabetic patients than adherence to individual nutrient-based recommendations [47]. These diet quality indices, the AHEI-2010, DQI-I and DASH score, represent dietary patterns that are generally characterized by a high intake of plant foods and a low intake of animal-based, high-fat and processed foods, although they differ in certain dietary components [13, 14, 19]. Thus, our results suggest that promoting adherence to largely plant-based dietary patterns and reducing energy intake are important for patient education and clinical practice in tackling obesity among Chinese T2D patients.

### Strengths and limitations

The findings of the present study should be interpreted in light of the study’s strengths and limitations. First, this is the first study to report an association between diet quality and obesity in Chinese adults with T2D. Another strength of this study is its detailed documentation of the study participants’ use of antidiabetic medication and their socio-demographic, clinical, and metabolic profiles, which were obtained from comprehensive diabetes metabolic assessments; this information enabled analyses to account for important confounding variables related to obesity. In addition, physical activity and dietary intake were assessed using locally validated questionnaires, the IPAQ-SF– Chinese version [26] and the FFQ [27], which were administered through face-to-face interviews by a single experienced dietitian using visual aids to minimize measurement errors. Furthermore, because all the study participants were also enrolled in the Hong Kong Diabetes Registry [10, 11], there was potential to investigate trends and associations among dietary quality, weight change, and other diabetes-related metabolic outcomes in a prospective follow-up of these study participants.

We acknowledge some limitations in the present study. First, the cross-sectional nature of this study limited the ability to suggest a causal relationship between diet quality and obesity. Second, subject recruitment was intentionally based on two distinct, non-continuous BMI ranges rather than on a continuum of BMI to enable potentially greater contrast between the two groups of T2D patients. Future studies using larger sample sizes with a continuum of weight classifications should be considered.

## Conclusions

Better diet quality, as quantified by AHEI-2010 scores, was significantly associated with lower odds of obesity in Chinese adults with T2D, although the relationship was weak. This suggests that other factors may influence the interplay between diet quality and obesity. A comprehensive approach with dietary patterns that reflect high consumption of plant-based foods, such as whole grains, vegetables and fruits, and low consumption of animal-based, high-fat and processed foods may be imperative for optimizing nutritional guidance in patient education and clinical practice for Chinese T2D patients, especially those with obesity. Our study findings suggest the need for healthcare professionals to formulate and implement dietary strategies that address both diet quality and energy reduction for obesity prevention and management in this population.

## Additional file

Additional file 1: **Table S1.** Scoring criteria of the Alternate Healthy Eating Index-2010 (AHEI-2010)^a^. **Table S2.** Scoring criteria of the Diet Quality Index-International (DQI-I)^a^. **Table S3.** Scoring criteria of the Dietary Approaches to Stop Hypertension (DASH)^a^ score. (DOCX 26 kb)

## Abbreviations

AHEI-2010: Alternate Healthy Eating Index-2010
BMI: Body mass index
CI: Confidence interval
DASH: Dietary Approaches to Stop Hypertension
DBP: Diastolic blood pressure
DQI-I: Diet Quality Index-International
FFQ: Food Frequency Questionnaire
FPG: Fasting plasma glucose
HbA_1C_: glycated hemoglobin
HDL-C: high-density lipoprotein cholesterol
IPAQ-SF: International Physical Activity Questionnaire - Short Form
LDL-C: Low-density lipoprotein cholesterol
MUFA: Monounsaturated fatty acids
OR: Odds ratio
PUFA: Polyunsaturated fatty acids
SBP: Systolic blood pressure
SFA: Saturated fatty acids
T2D: Type 2 diabetes
TC: Total cholesterol
TG: Triglycerides
WHO: World Health Organization
