# Supplementary material for: Diet quality is inversely associated with obesity in Chinese adults with type 2 diabetes
Source: Nutr J. 2018 Jul 3;17:63. doi: 10.1186/s12937-018-0374-6 (PMC6031190; doi:10.1186/s12937-018-0374-6)
Supplement: Supplementary file 1 — Table S1. Scoring criteria of the Alternate Healthy Eating Index-2010 (AHEI-2010)a. Table S2. Scoring criteria of the Diet Quality Index-International (DQI-I)a. Table S3. Scoring criteria of the Dietary Approaches to Stop Hypertension (DASH)a score. (DOCX 26 kb) [file 12937_2018_374_MOESM1_ESM.docx]

**Table S1.** Scoring criteria of the Alternate Healthy Eating Index-2010 (AHEI-2010)^a^.

|  | Criteria for scoring of each component | | | | | |
| --- | --- | --- | --- | --- | --- | --- |
|  | 0  (Minimum point) | 2 | 4 | 6 | 8 | 10  (Maximum point) |
| Vegetables^b^, servings/day | 0 | >0 | ≥1.25 | ≥2.5 | ≥3.75 | ≥5 |
| Fruits^c^,  servings/day | 0 | >0 | ≥1 | ≥2 | ≥3 | ≥4 |
| Whole grains, g/day | 0 | M: >0  F: >0 | M: ≥22.5  F: ≥18.75 | M: ≥45  F: ≥37.5 | M: ≥67.5  F: ≥56.25 | M: 90  F: 75 |
| Sugar-sweetened beverages and fruit juice^d^, servings/day | ≥1 | ≥0.75 | ≥0.5 | ≥0.25 | >0 | 0 |
| Nuts and legumes^e^, servings/day | 0 | >0 | ≥0.25 | ≥0.5 | ≥0.75 | ≥1 |
| Red/processed meat^f^, servings/day | ≥1.5 | ≥1.125 | ≥0.75 | ≥0.375 | >0 | 0 |
| Trans fat, % of total energy | ≥4 | ≥3.125 | ≥2.25 | ≥1.375 | >0.5 | ≤0.5 |
| Long-chain omega-3 fats (EPA+DHA), mg/day | 0 | >0 | ≥62.5 | ≥125 | ≥187.5 | 250 |
| PUFA, % of total energy | ≤2 | >2 | ≥4 | ≥6 | ≥8 | ≥10 |
| Sodium, mg/day | Highest decile  M: <2665  F: <2153 | M: ≥2665  F: ≥2153 | M: ≥3176  F: ≥2710 | M: ≥3687  F: ≥3266 | M: ≥4198  F: ≥3823 | Lowest decile  M: ≥4709  F: ≥4379 |
| Alcohol^g^, drinks/day | M: ≥3.5  F: ≥2.5 | M: <0.5 or >2.0-3.4 for 2.5 points  F: <0.5 or >1.5-2.4 for 2.5 points | | | | M: 0.5-2.0  F: 0.5-1.5 |

^a^Total score ranges from 0-110 points. A higher total score indicates a higher diet quality.

^b^1 serving = 0.5 cup of vegetables or 1 cup of green leafy vegetables.

^c^1 serving = 1 medium piece or 0.5 cup of fruit.

^d^1 serving = 8 oz sugar-sweetened beverages and fruit juice (1 oz = 28.35 g).

^e^1 serving = 1 oz nuts and legumes (1 oz = 28.35 g).

^f^1 serving = 4 oz red meat or 1.5 oz processed meat (1 oz = 28.35 g).

^g^1 drink = 4 oz of wine, 12 oz of beer, or 1.5 oz of liquor (1 oz = 28.35 g).

DHA: docosahexaenoic acid, EPA: eicosapentaenoic acid, PUFA: polyunsaturated fatty acids, M: male, F: female.

**Table S2.** Scoring criteria of the Diet Quality Index-International (DQI-I)^a^.

| Component | Point | Scoring criteria |
| --- | --- | --- |
| **Variety** | **0–20 points** |  |
| Overall food group variety (meat/poultry/fish/eggs; dairy/beans; grain; fruit; vegetable)^b,c,d,e,f^ | 0–15 points | ≥1 serving from each food group/d = 15 points |
|  |  | Any 1 food group missing/d = 12 points |
|  |  | Any 2 food groups missing/d = 9 points |
|  |  | Any 3 food groups missing/d = 6 points |
|  |  | ≥4 food groups missing/d = 3 points |
|  |  | None of any food groups = 0 point |
| Within-group variety for protein source (meat, poultry, fish, dairy, beans, eggs) | 0–5 points | ≥3 different sources/d = 5 points |
|  |  | 2 different sources/d = 3 points |
|  |  | From 1 source/d = 1 point |
|  |  | None = 0 point |
| **Adequacy** | **0–40 points** |  |
| Vegetable^f^ | 0–5 points | ≥3–5 servings/day = 5 points, 0 serving/day = 0 point |
| Fruit^e^ | 0–5 points | ≥2–4 servings/day= 5 points, 0 serving/day = 0 point |
| Grain^d^ | 0–5 points | ≥6–11 servings/day = 5 points, 0 serving/day = 0 point |
| Fiber | 0–5 points | ≥20–30 g/day = 5 points, 0 g/day = 0 point |
| Protein | 0–5 points | ≥10% of total energy/day = 5 points, 0% of total energy/day = 0 point |
| Iron^h^ | 0–5 points | ≥100% RNI/day = 5 points, 0% RNI/day = 0 point |
| Calcium^i^ | 0–5 points | ≥100% AI/day = 5 points, 0% AI/day = 0 point |
| Vitamin C^j^ | 0–5 points | ≥100% RNI/day = 5 points, 0% RNI/day = 0 point |
| **Moderation** | **0–24 points** |  |
| Total fat | 0–6 points | ≤20% of total energy/day = 6 |
|  |  | >20–30% of total energy/day = 3 |
|  |  | >30% of total energy/day = 0 |
| Saturated fat | 0–6 points | ≤7% of total energy/day = 6 |
|  |  | >7–10% of total energy/day = 3 |
|  |  | >10% of total energy/day = 0 |
| Cholesterol | 0–6 points | ≤300 mg/day = 6 |
|  |  | >300–400 mg/day = 3 points |
|  |  | >400 mg/day = 0 points |
| Sodium | 0–6 points | ≤2400 mg/day = 6 points |
|  |  | >2400–3400 mg/day= 3 points |
|  |  | >3400 mg/day = 0 points |
| **Overall balance** | **0–10 points** |  |
| Macronutrient ratio^k^ (carbohydrate: protein: fat) | 0–6 points | 55-65: 10-15: 15-25 = 6 points |
|  |  | 52-68: 9-16: 13-27 = 4 points |
|  |  | 50-70: 8-17: 12-30 = 2 points |
|  |  | Otherwise = 0 point |
| Fatty acid ratio (PUFA: MUFA: SFA) | 0–4 points | P/S = 1-1.5 and M/S = 1-1.5 = 4 points |
|  |  | Else if P/S = 0.8-1.7 and M/S = 0.8-1.7 = 2 points |
|  |  | Otherwise = 0 point |

^a^ Total score ranges from 0-94 points. A higher score indicates a higher overall diet quality.

^b^1 serving = 1 oz meat/poultry/fish/eggs/tofu (1 oz=28.35 g).

^c^1 serving = 250 ml milk/ 30 g milk powder/ 40 g cheese/ 150 ml yogurt.

^d^1 serving = 200g congee/ 100g rice or pasta/ 50g bread.

^e^1 serving = 1 medium piece or 0.5 cup of fruit.

^f^1 serving = 0.5 cup of vegetables or 1 cup of green leafy vegetables.

^g^ ≥8 servings of grains for men or ≥6 servings for women = 5 points.

^h^ Chinese RNI (individuals aged 50-64 years) = 12 mg/d iron.

^I^ Chinese AI (individuals aged 50-64 years) = 1000 mg/d calcium.

^j^ Chinese RNI (individuals aged 50-64 years) = 100 mg/d vitamin C.

^k^ Ratio of energy from carbohydrate to protein to fat.

AI: Adequate Intake, MUFA: monounsaturated fatty acids, M/S: ratio of monounsaturated fatty acids to saturated fatty acids intake, PUFA: polyunsaturated fatty acids, P/S: ratio of polyunsaturated fatty acids to saturated fatty acids intake, RNI: Recommended Nutrient Intake, SFA: saturated fatty acids.

**Table S3.** Scoring criteria of the Dietary Approaches to Stop Hypertension (DASH)^a^ score.

| Component | Point | Scoring Criteria |
| --- | --- | --- |
| Fruits | Q1 = 1 point  Q2 = 2 points  Q3 = 3 points  Q4 = 4 points  Q5 = 5 points | Q1 = 81.9 g (0.5 serving)  Q5 = 227.4 g (1.5 servings) |
| Vegetables |  | Q1 = 85.5 g (1.1 servings)  Q5 = 232.9 g (3.1 servings) |
| Nuts and legumes |  | Q1 = 11.2 g (1.1 servings)  Q5 = 232.9 g (3.1 servings) |
| Sodium | Q1 = 5 points  Q2 = 4 points  Q3 = 3 points  Q4 = 2 points  Q5 = 1 point | Q1 = 2725 mg  Q5 = 4139 mg |
| Red and processed meat |  | Q1 = 57.8 g (2.0 servings)  Q5 = 176.8 g (6.2 servings) |
| Whole grains | Zero intake = 1 point  Q1 = 2 points  Q2 = 3 points  Q3 = 4 points  Q4 = 5 points | Q1 = 57.0 g  Q4 = 114.0 g |
| Low-fat dairy |  | Q1 = 30.0 ml |
|  |  | Q4 = 150.0 ml |
| Sweetened beverages | Zero intake = 5 points  Q1 = 4 points  Q2 = 3 points  Q3 = 2 points  Q4 = 1 point | Q1 = 35.6 ml  Q4 = 250.0 ml |

^a^ Total score ranges from 8-40 points. A higher total score indicates a higher diet quality.

Q: quartile or quintile.
